# Islet transplantation improved penile tissue fibrosis in a rat model of type 1 diabetes

**DOI:** 10.1186/s12902-018-0276-9

**Published:** 2018-07-27

**Authors:** Zhigang Wu, Hongwei Wang, Fubiao Ni, Xuan Jiang, Ziqiang Xu, Chengyang Liu, Yong Cai, Hongxing Fu, Jiao Luo, Wenwei Chen, Bicheng Chen, Zhixian Yu

**Affiliations:** 1 Department of Andrology, The First Affiliated Hospital of Wenzhou Medical University, Wenzhou, 325000 Zhejiang Province China; 2 Hepatobiliary and pancreatic surgery laboratory, The First Affiliated Hospital of Wenzhou Medical University, Wenzhou, 325000 Zhejiang Province China; 3 Department of Transplantation, The First Affiliated Hospital of Wenzhou Medical University, Wenzhou, 325000 Zhejiang Province China; 4 School of Pharmacy, Wenzhou Medical University, Wenzhou, 325000 Zhejiang Province China; 5 Department of Surgery, Perelman School of Medicine at the University of Pennsylvania, Philadelphia, PA 19104-5160 USA; 6 Department of Urology, the First Affiliated Hospital of Wenzhou Medical University, Wenzhou, 325000 Zhejiang Province China

**Keywords:** Diabetes mellitus, Erectile dysfunction, α-SMA, TGF-β1

## Abstract

**Background:**

Glycaemic control is one of the most effective strategies for the treatment of diabetes-related erectile dysfunction (DMED). Compared to conventional anti-diabetic drugs and insulin, islet transplantation is more effective in the treatment of diabetic complications. The aim of this study was to investigate the efficacy of islet transplantation for reversing advanced-stage DMED in rats and to observe its influence on corpus cavernosum fibrosis.

**Methods:**

Wistar rats were intraperitoneally injected with streptozotocin to establish a diabetes model. After 12 weeks, the rats were divided into 4 groups: diabetic, insulin, islet transplantation, and normal control. Following supplementation, the changes in blood glucose and weight were determined sequentially. Penile erectile function was evaluated by apomorphine experiments in the fourth week, and the penile corpus cavernosum was also collected for assessment by Masson staining, immunohistochemistry and Western blot to observe the spongy tissue and the related cellular changes at the molecular level.

**Results:**

Islet transplantation significantly ameliorated penile erectile function in advanced-stage diabetic rats. The ratio of corpus cavernosum smooth muscle cells to fibroblasts and the expression level of α-SMA in the islet transplantation group were significantly higher than those in the diabetic and insulin groups. In addition, the expression levels of TGF-β1, p-Samd2, and connective tissue growth factor (CTGF) in the islet transplantation and insulin groups were much lower than those in the diabetic group, while those in the islet transplantation group were significantly lower than those in the insulin group.

**Conclusions:**

Our findings strongly suggest that islet transplantation can promote the regeneration of smooth muscle cells and ameliorate corpus cavernosum fibrosis to restore its normal structure in advanced-stage diabetic rats. The possible mechanism of ameliorating corpus cavernosum fibrosis by islet transplantation may be associated with improvement of the hyperglycaemic status in diabetic rats, thereby inhibiting the TGF-β1/Samd2/CTGF pathway.

## Background

Clinically, diabetes mellitus is one of the most common causes of erectile dysfunction (ED) [1]. The incidence of ED in patients with diabetes is 4 times that in non-diabetic patients. Approximately 50–75% of male diabetic patients have ED, and the condition usually occurs during the early stage of diabetes [2]. Long-term hyperglycaemia not only causes corpus cavernosum blood vessel, nerve and endothelial dysfunction but also leads to penile tissue fibrosis, which damages the structure of the corpus cavernosum and decreases erectile function [3, 4]. Phosphodiesterase type 5 (PDE5) inhibitors are currently one of the most important practical treatment choices for ED; PDE5 inhibitors up-regulate the NO-cGMP pathway to improve vascular endothelial function and promote penile erection [5, 6]. However, patients with diabetes-related ED (DMED) often respond poorly to PDE5 inhibitors, potentially due to apoptosis of cavernosum smooth muscle cells (SMCs) and proliferation of cavernosum fibrous tissue [7]. Among a variety of profibrotic factors, transforming growth factor-β1 (TGF-β1) has been regarded as the fibrogenic cytokine most closely related to cavernosum fibrosis [8].

In the early stages, strict glycaemic control is an effective strategy for inhibiting the progression of DMED [9]. Kwon et al. reported that erectile function in diabetic rats can be recovered to near normal levels by tightly controlling blood sugar levels in the early stage of diabetes [10]. However, in other stages, insulin treatment or drug therapy cannot achieve satisfactory results [11]. In addition, maintaining strict glycaemic control in every diabetic patient is almost impossible in clinical practice.

Pancreatic transplantation and islet cell transplantation are currently the most effective methods for clinical treatment of various chronic complications that are associated with diabetes [12]. Compared to pancreatic transplantation, islet transplantation is a simpler operation with a lower risk of morbidity. In addition, recipients can obtain additional donor islet cells through repeated surgeries,to fully restore blood glucose levels in diabetic patients to normal levels [13, 14]. Recent studies have shown that pancreas or islet transplantation can ameliorate and even reverse diabetic complications, including nephropathy, retinopathy and neuropathy in the early stage [15–17]. However, the effect of islet transplantation on DMED has not been reported.

In the present study, we further investigated the significance of the recovery of penile structure and function in advanced-stage DMED rats treated with islet transplantation. We also discussed the associations between the TGF-β1/Smad2 pathway and corpus cavernosum fibrosis.

## Methods

### Animal model and groups

A total of 42 male Wistar rats weighing 200–220 g were provided by the Experimental Animal Center of Wenzhou Medical University. All rats were housed with a 12-h light/dark cycle at 24 °C ± 1 °C and fed ad libitum for 1 week before starting the study. All animal experiments were approved by the Zhejiang Management Committee for Medical Laboratory Animal Sciences. Diabetic rat models were generated by a single intraperitoneal injection of streptozotocin (50 mg/kg of body weight) in sodium citrate buffer (pH 4.5) after an overnight fast. Plasma glucose concentrations were measured in a drop of tail vein blood using an Accu-Chek glucometer (Roche Diagnostics, Indianapolis, IN). Seven days later, a non-fasted blood glucose concentration ≥ 16.67 mmol/l for 3 days indicated the successful establishment of the rat experimental diabetic model. Twelve weeks after the induction of diabetes, rats were randomly divided into four different groups: diabetes-related erectile dysfunction group (ED group, *n* = 6), these rats were left untreated and studied 4 weeks later; islet transplantation group (IT group, *n* = 6), these rats underwent islet transplantation under the left kidney capsule and were studied 4 weeks later; insulin treatment group (INS group, *n* = 6), these rats were given insulin (WanBang Pharmaceuticals, JiangSu, China) by subcutaneous injection at 9 a.m. and 9 p.m. every day for 4 weeks (3 U per injection); and a normal control group (control group, *n* = 6).

### Islet transplantation

Islets were isolated from rat pancreases using a procedure previously described by our laboratory [18]. Islets from three donor rats were supplied for each recipient. The same procedure was followed for each rat. Briefly, rats were anaesthetized by intraperitoneal injection of chloral hydrate. Then, a laparotomy was performed to expose the pancreas. The location at which the common bile duct meetsthe intestine was determined and ligated, and 8 ml of collagenase V (0.8 mg/ml, dissolved in Hank’s solution) was injected into the common bile duct by retrograde intubation. When the pancreas was fully inflated, it was separated from the surrounding tissues with forceps, transferred into a 50-ml centrifuge tube and digested for 10–15 min at 37 ± 0.5 °C. After digestion, the tissue was washed with Hank’s solution three times. Then, the islets were purified by density gradient solutions (Histopaque-1119 and Histopaque-1077) and centrifuged at 2000 rpm for 5 min. The supernatant was poured into a new centrifuge tube and transferred to a black glass culture dish for manual selection. The final purified islets were cultured in RPMI-1640 (Gibco, Carlsbad, CA, USA) containing 10% foetal bovine serum (FBS) (Gibco, Invitrogen, Inc., USA) at 37 °C in 5% CO_2_. The purified islets were adjusted to appropriate concentrations in the culture medium and transferred to a small culture dish with a 2-mm lattice for counting under a microscope. According to Lembert and others, the cell clusters were counted, and the diameters were measured with a microscope eyepiece scale. The total islet equivalents (IEQs) were calculated according to the IEQ calculation formula. A single aliquot of 100 freshly isolated islets was aspirated into a 200-μl pipette tip and transferred to a small culture dish. Propidium iodide (PI) and fluorescein diacetate (FDA) were added to evaluate islet activity by FDA-PI staining under an inverted fluorescence microscope. The activity ratio of 100 islet cell clusters was determined to estimate the total number of purified islets. From the final purified islets, approximately 800–1000 IEQs were aspirated into a 1-ml syringe connected to P-50 polyethylene tubing, and the islets were transferred to the head end of the kidney. The recipient rat was anaesthetized by intraperitoneal injection of chloral hydrate; the left flank was shaved, and the kidney was exposed through a small lumbar incision. Capsulotomy of the kidney was performed on the caudal outer surface, and the tip of the polyethylene tubing was inserted and advanced gently under the kidney capsule. The surface of the kidney was kept moist with saline during the procedure. The islets in the tubing were pushed out slowly and carefully, and the tube was removed when the islets were transferred into the capsule. Then, the kidney was gently replaced into the peritoneum, haemostasis was performed by compression with a cotton swab, and the incision was sutured layer by layer.

### Evaluation of erectile function

According to the method reported by Heaton [19], rats in each group were placed in the observation cage after feeding. The room lights were dimmed to a level that allowed observation, and the interior environment was kept quiet, allowing the rats to adapt to the environment for 10 min. Then, apomorphine (APO) was injected in the relaxed neck skin of the rat (150 μg/kg, APO dissolved in 1 mg/kg of vitamin C and physiological saline, with the volume adjusted to 5 ml/kg); each rat was recorded with a video camera during the first 30 min after injection, and the number of penile erections was observed and recorded. Penile erection was indicated by the appearance of the glans penis at the end of the penis. Each rat in each group was tested six times, and the percentage of all rats in the group with erectile function was recorded as the erectile rate.

### Histological and immunohistochemical examinations

Rat cavernosum was dissected and fixed with 4% formalin. The tissue in the paraffin block was sliced to a thickness of 5 μm for immunohistochemical staining. To detect the ratio of rat cavernosum muscle to collagen, cavernosum tissue sections were stained with Masson’s trichrome stain. For immunohistochemical staining, the slides were incubated overnight with antibodies against caspase-3(CST, 1:200), α-SMA (Abcam, 1:200). The tissue was then incubated with goat anti-rabbit antibody, visualized with diaminobenzidine (DAB, brown colour, ZSGB-BIO, Beijing, China) and analysed with Image-Pro Plus 6.0.

### Western blot analysis

The proteins were extracted from the corpus cavernosum and quantified by BCA protein assay (Beyotime, Shanghai, China). The protein extract was electrophoresed on sodium dodecyl sulphatepolyacrylamide gel andtransferred to a polyvinylidene fluoride film, which was blocked with 5% skimmed milk at room temperature for 1 hour. The membrane was incubated overnight at 4 °C with a primary antibody: TGF-β1 (CST, 1:1000), p-Smad2 (Abcam, 1:500), Smad2 (Abcam, 1:500) and CTGF (CST, 1:1000) and then incubated with horseradish peroxidase-conjugated secondary antibody at room temperature for 2 hours. Finally, an electrochemical luminescence system (Amersham, Arlington Heights, IL, USA) was used for visualization.

### Statistical analysis

All statistical data are expressed as the mean ± standard deviation. The differences among groups were analysed using one-way ANOVA; a value of *P* < 0.05 was considered statistically significant. GraphPad Prism 5.0 was used for the statistical analyses.

## Results

### Evaluation of islet activityand islet cells under the renal capsule

**Fig. 1:**
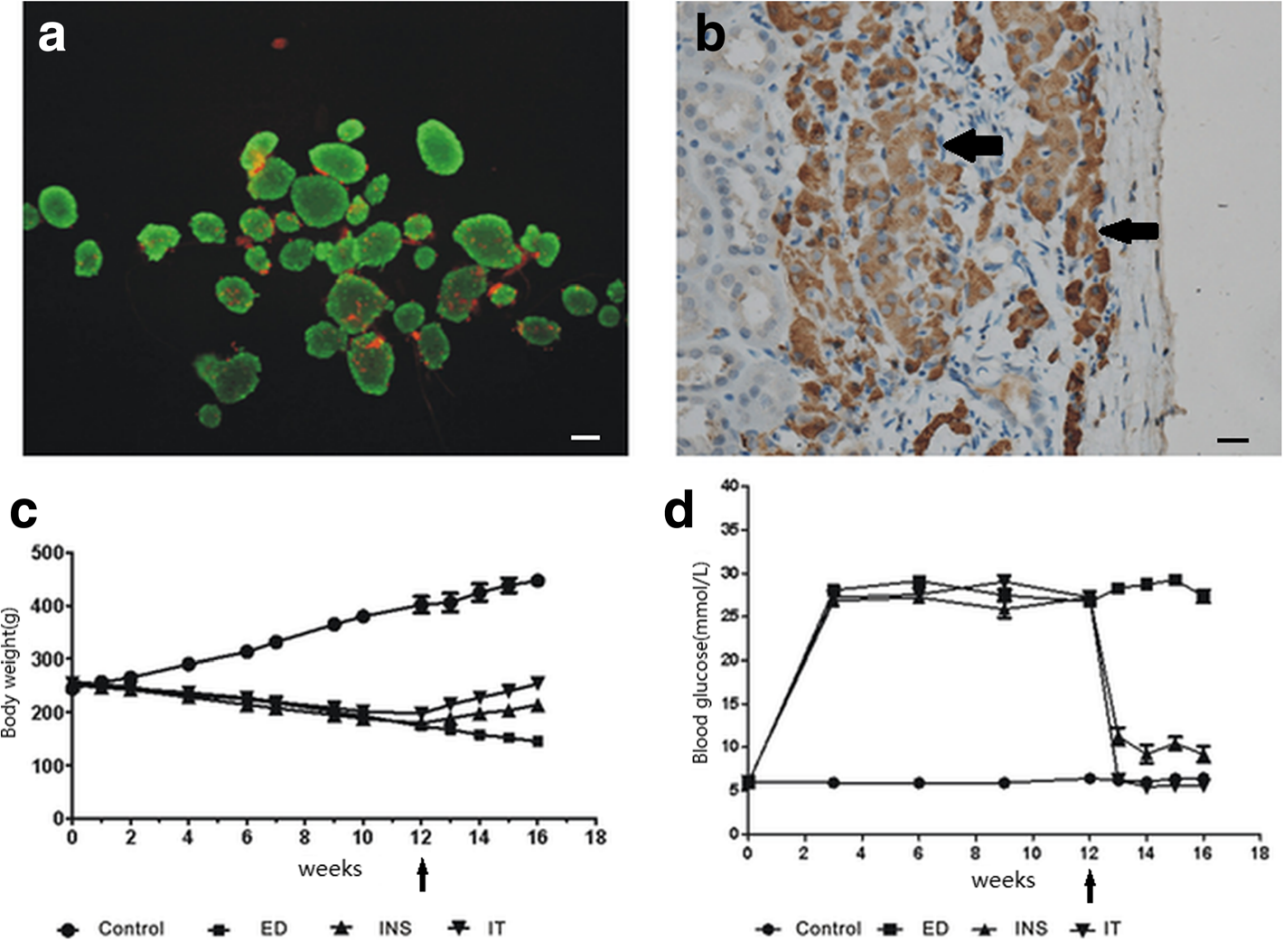
Evaluation of islet activity and body weights and blood glucose levels over 16 weeks. **a** Evaluation of activity in the isolated islets (FDA-PI staining, × 100). Bar = 25 μm. **b** Immunohistochemical staining for insulin as brown areas, which stains transplanted islets under the kidney capsule (magnification × 200). Bar = 25 μm. **c** Body weight changes over 16 weeks and the arrow indicates 12 weeks as the start of treatment. **d** Nonfasting blood glucose levels for each group over 16 weeks and the arrow indicates 12 weeks as the start of treatment. Model group: diabetes-related erectile dysfunction rat models established at 12 weeks. Control = normal control, ED = diabetes-related erectile dysfunction, INS = insulin treatment, IT = islet transplantation

As shown in Fig. 1a, islet activity was evaluated by FDA-PI staining with an aliquot of islets before transplantation, and the results revealed a high level of islet activity. Transplanted islet cells under the renal capsule showed high activity by immunohistochemical examination for insulin 4 weeks after transplantation (Fig. 1b).

### General characteristics of diabetic rats

Diabetic rats treated with insulin or islet transplantation showed a significant decrease in blood glucose levels and increase in body weight compared to rats that were not treated. Islet transplantation treatment significantly improved the blood glucose level and body weight compared to insulin treatment (Fig. 1c and d).

### Islet transplantation restored erectile function

**Fig. 2:**
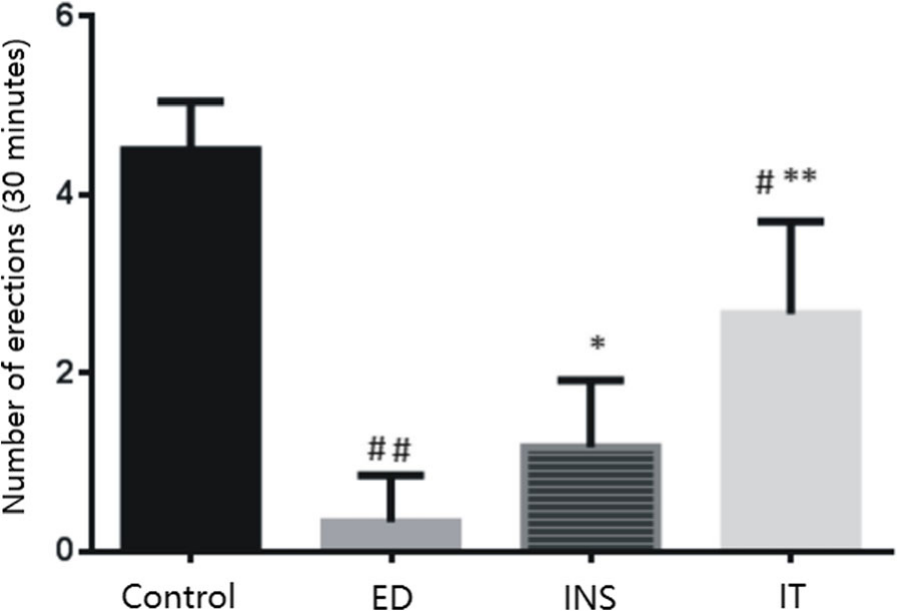
Evaluation of erectile function. Apomorphine experiments are performed to evaluate erectile function in each group. Values are presented mean ± standard deviation of the mean and the differences among groups are analysed using one-way ANOVA; ^**^P < 0.01 vs. the Control group; ^*^P < 0.05and^**^P < 0.01vs. the ED group;^#^*P <* 0.01 vs. the INS group. Control = normal control, ED = diabetes-related erectile dysfunction, INS = insulin treatment, IT = islet transplantation

After injection with APO, the erectile response in diabetic rats was significantly lower than that in control rats. The erectile response was significantly improved in the islet transplantation and insulin groups compared to the diabetic group after subcutaneous injection of APO, but the effect of islet transplantation treatment was greater than that of insulin treatment (Fig. 2).

### Islet transplantation up-regulated the smooth muscle/collagen ratio and α-SMA protein expression in the corpus cavernosum

**Fig. 3:**
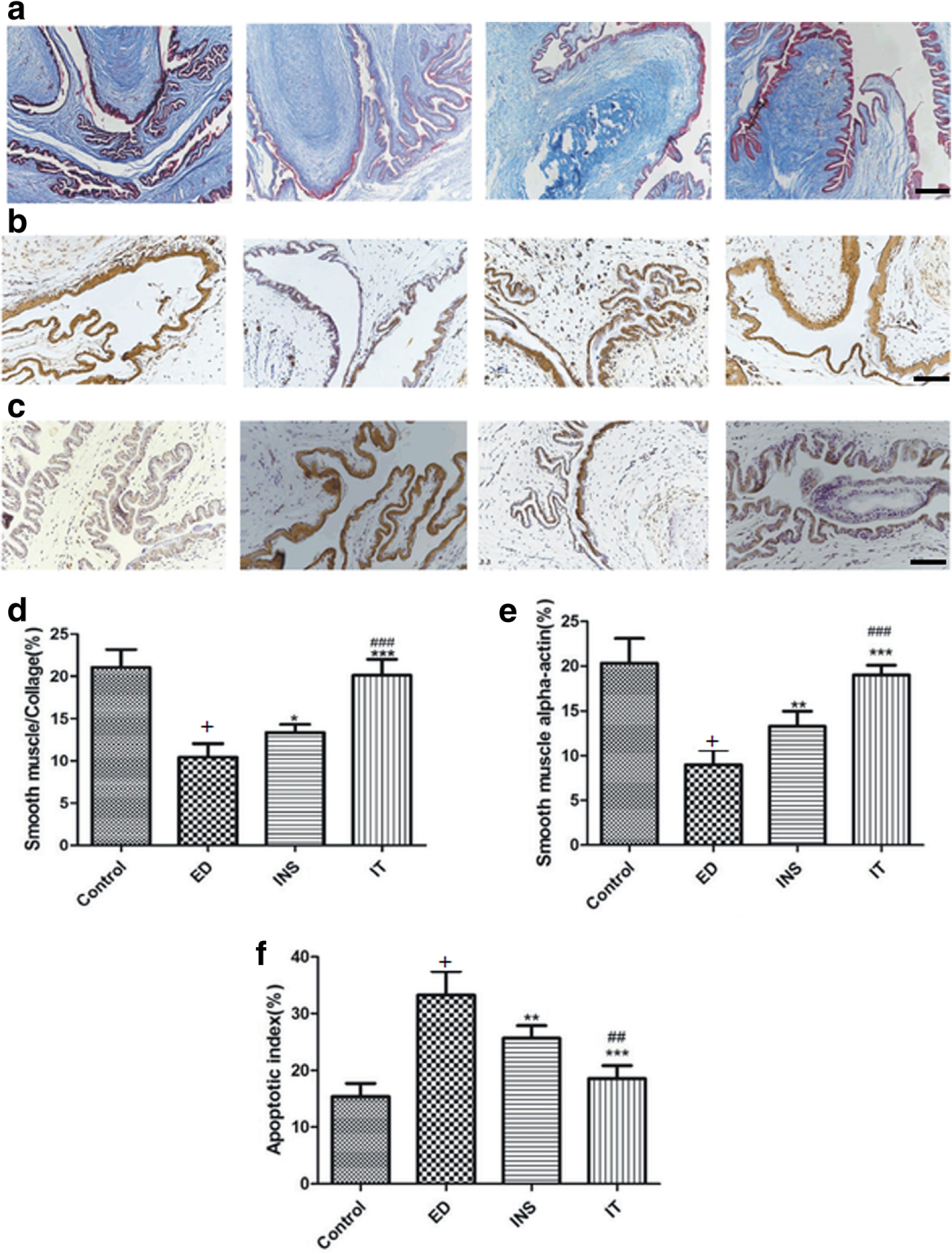
Masson’s trichrome staining and immunohistochemical staining. **a** Penis samples are prepared for the detection of corpus cavernosum tissue fibrosis using Masson’s trichrome staining (magnification × 100). The smooth muscle components appeared red colour. The collagen components appear blue colour. Bar = 25 μm. **b** Immunohistochemical staining for α-SMA as brown areas, which stains smooth muscle cell(SMC) in the corpus cavernosum (magnification × 200). Bar = 50 μm. **c** Immunohistochemical staining for caspase-3 as brown areas, which stains SMCs apoptosis in the corpus cavernosum(magnification × 200). Bar = 50 μm. **d** Semiquantitative image analysis of muscle/collagen ratio in corpus cavernosum tissues was performed using GraphPad Prism 5.0 soft. **e** Semiquantitative image analysis of α-SMA expression in corpus cavernosum. **f** Apoptotic index presented as the ratio of apoptotic SMCs (expression of caspase-3) to the total SMCs in corpus cavernosum. Values are presented mean ± standard deviation of the mean and the differences among groups are analysed using one-way ANOVA; +*P* < 0.001 vs. the Control group; *P < 0.05, **P < 0.01, and ***P < 0.001 vs. the ED group; ## *P* < 0.01 and ### *P* < 0.001 vs. the INS group. Control = normal control, ED = diabetes-related erectile dysfunction, INS = insulin treatment, IT = islet transplantation

Compared to the control group, the smooth muscle/collagen ratio and expression level of α-SMA were significantly decreased in untreated diabetic rats. Insulin treatment significantly improved these parameters compared to no treatment among diabetic rats, but the effect of islet transplantation treatment was better than that of insulin or no treatment. No significant difference was observed in these parameters between the islet transplantation and control groups (Fig. 3a and b).

### Islet transplantation reduced diabetes-induced apoptosis in the corpus cavernosum

Compared to the control group, the number of capase-3-positive cells in the diabetic group was significantly increased. No significant difference was observed in the number of caspase-3-positive cells between the islet transplantation and control groups. Insulin also significantly improved this parameter, but the effect was less than that of islet transplantation (Fig. 3c).

### Islet transplantation inhibited diabetes-induced activation of the TGF-β1 signalling pathway

**Fig. 4:**
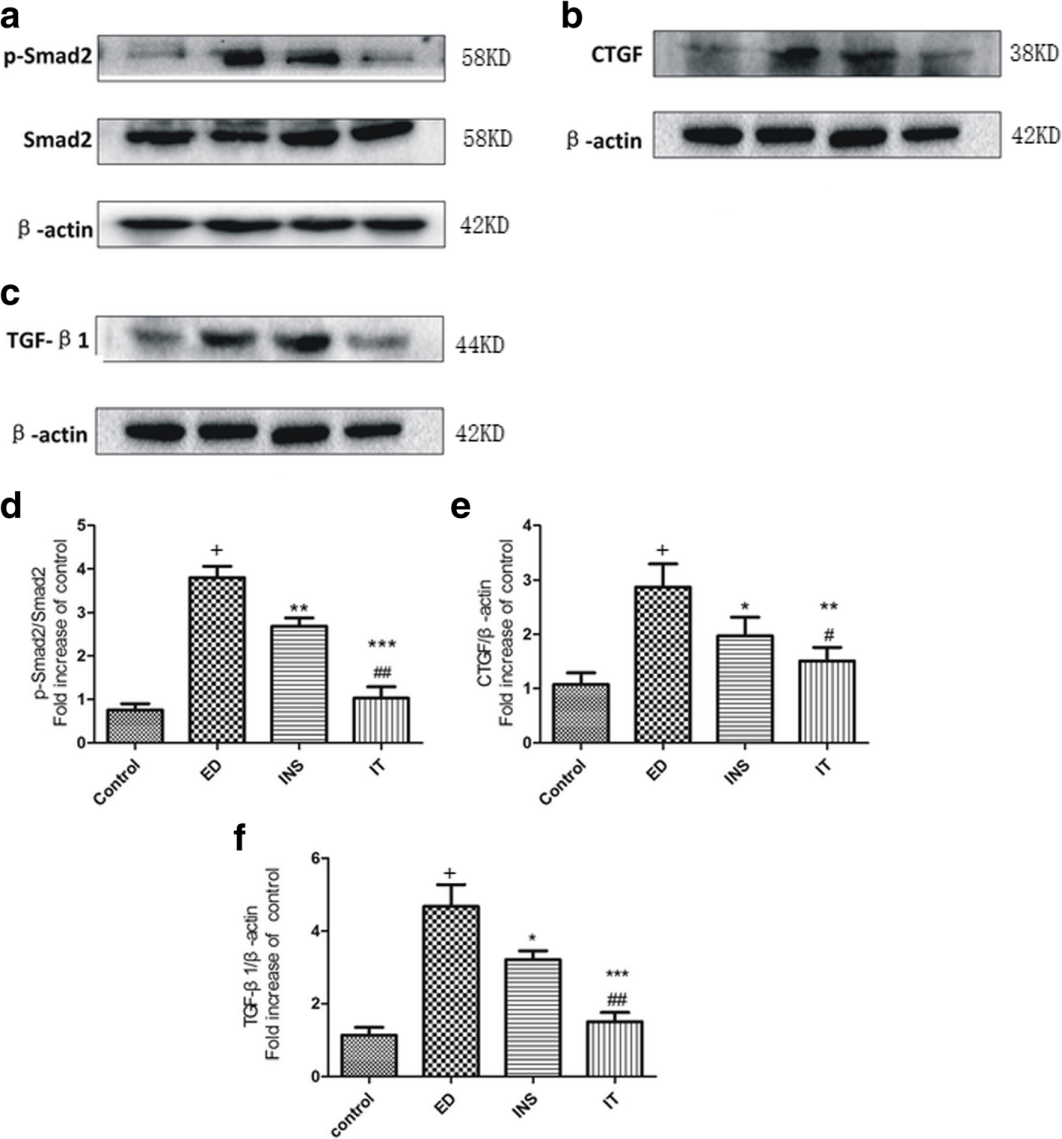
Western blot analysis. **a** Western blot analysis showing the protein expression levels of P-Smad2 and Smad2in rat corpus cavernosum tissues. **b** The expression of CTGF is measured by Western blot in rat corpus cavernosum tissues. **c** The expression of TGF-β1 is measured by Western blot in rat corpus cavernosum tissues. **d** Data are presented as the relative density of phospho-Smad2 compared with that of total Smad2. **e**-**f** β-actin is used as loading control and data are presented as the relative density of CTGF and TGF-β1 compared with that of β-actin. Values are presented mean ± standard deviation of the mean and the differences among groups are analysed using one-way ANOVA; ^+^*P* < 0.001 vs. the Control group; *P < 0.05 and **P < 0.01 vs. the ED group; ^#^*P* < 0.05 and ^##^*P* < 0.01 vs. the INS group.Control = normal control, ED = diabetes-related erectile dysfunction, INS = insulin treatment, IT = islet transplantation

As shown in Fig. 4, the expression levels of TGF-β1, p-Smad2 and CTGF after treatment with islet transplantation and insulin were significantly decreased compared tono treatment in the diabetic rats. However, treatment with islet transplantation suppressed these parameters more significantly than treatment with insulin; no statistically significant difference was found between the islet transplantation and control groups.

## Discussion

Long-term maintenance of stable and normal blood glucose levels is a key strategy for the treatment of diabetic ED. Over the past few decades, several studies have shown that pancreas or islet transplantation is an effective method to control blood glucose. Our study was designed to investigate the efficacy of islet transplantation for reversing advanced-stage DMED in rats and to establish whether this treatment is superior to insulin therapy for treating DMED in rats.

In this study, we confirmed that islet transplantation treatment significantly restored erectile function and penile structure in advanced-stage DMED rats. However, the therapeutic effect of insulin was unsatisfactory for recovering erectile function and penile structure. Furthermore, the present study showed that the detailed mechanisms of islet transplantation were SMC regeneration and structural integrity recovery in penile corpus cavernosum via inhibition of the TGF-β1/Smad2/CTGF pathway.

Although oral PDE5 inhibitors are highly efficacious and safe modalities for ED, patients with DMED frequently respond poorly to these drugs [20]. Strict glycaemic control is still a basic treatment of DMED. Moreover, the effect of glycaemic control is significantly affected by the degree of morbidity and treatment. A previous study showed that early treatment with insulin can reverse early erectile functional changes [9]. In contrast, another study reported that the degree of glycaemic control is likely to be more important than the timing of the initiation of glycaemic control for treating DMED [11]. Clinically, various types of insulin preparations are the most widely used measures of blood sugar control. To date, however, the application of insulin to diabetic patients for strict glycaemic control is limited because patients must manage the risk of hypoglycaemic shock. Furthermore, insulin therapy cannot significantly improve diabetes-related complications in patients with advanced diabetes mellitus. No recovery of penile cavernosum was observed when insulin treatment started later than 12 weeks after inducing diabetes mellitus in rats [21]. Islet transplantation is the most effective technique for restoring normal blood glucose levels. Yajun Ruan et al. used an APO experiment to detect penile erectile function in 12-week diabetic rats and found that the APO test was negative and used it as DMED. In addition, FENG ZHOU et al. also demonstrated that 12 weeks were sufficient to cause penile erectile dysfunction in diabetic rats [22, 23]. Therefore, in our study, the advanced-stage DMED rat model was established at 12 weeks after inducing diabetes mellitus. Insulin or islet transplantation was administered continuously in the rat model for 4 weeks. Throughout the experiment, islet transplantation could maintain the blood glucose levels of DMED rats in the normal range. However, insulin treatment was not able to maintain stable blood glucose levels. Furthermore, SMCs in the cavernosum account for 40–52% of cells and maintain penile contractility [24]. Chronic hyperglycaemia induces the loss of SMCs and leads to fibrous-muscular changes in penile tissue [25, 26]. Our studies have shown that penile erectile function is improved in advance-stage diabetic rats treated with insulin compared to rats in the DM group; however, the loss of SMCs and the increase in fibrous tissue in the corpus cavernosum were more notable in the insulin group than in the control group. In our study, penile tissues from the islet transplantation group exhibited significantly higher smooth muscle/collagen ratios and higher α-SMA expression levels in the corpus cavernosum than the insulin and DM groups. α-SMA, a typical isoform found in SMCs, is used to evaluate smooth muscle content in the corpus cavernosum [27]. Our study also confirmed that SMC apoptosis was significantly lower in the islet transplantation group than in the insulin and DM groups. Our results suggest that the cavernous structure in the islet transplantation group was similar to the normal structure of the penile sponge. Insulin treatment could delay only fibrosis of the corpus cavernosum, but islet transplantation could promote SMC regeneration, improve the penile cavernosum structure, and ultimately restore erectile function in advanced-stage DMED rats.

Expression of TGF-β1 and its downstream effectors in penile tissues was significantly increased in diabetic rats. TGF-β1 regulates fibrotic effects via activating receptor-associated Smad2 and Smad3, which translocate to the nucleus and modulate the transcription of TGF-β1 responsive genes [28]. TGF-β1 may decrease the elasticity and compliance of the penis by changing the collagen types and increasing collagen synthesis [29]. Evidence indicates that TGF-β1 signalling promotes SMC apoptosis and inhibits the regenerative capacity of SMCs in the penis [30]. CTGF, which is regulated by TGF-β1, is a vital profibrotic molecule in the tissue. Overexpression of CTGF induces fibroblast proliferation, migration, and adhesionextracellular matrix overexpression, and SMC apoptosis. Researchers have also shown that up-regulation of the TGF-β1/Smad2/CTGF pathway might play a key role in fibrosis induction in the penile tissue of diabetic rats [31]. Our studies confirmed that the TGF-β1/Smad2 and p-Smad2/CTGF pathways in the corpus cavernosum were significantly activated in advanced-stage DMED rats. Maintaining adequate blood glucose levels is the most effective measure to inhibit the TGF-β1 signalling pathway in diabetic rats. In this study, we performed immunohistochemistry and Western blotting to determine whether islet transplantation can significantly suppress the expression levels of TGF-β1, p-Smad2 and CTGF in advanced-stage DMED rats compared to those in the other groups. These results are consistent with our previous studies in which islet transplantation could decrease the expression levels of fibrotic factors including TGF-β1 and CTGF, in early diabetic nephropathy. This result may provide a better explanation for the molecular mechanism of islet transplantation in recovering erectile function and the penile cavernosum structure.

There were some limitations in the present study. First, diabetic autonomic neuropathy and endothelial dysfunction are also involved in DMED. Thus, additional studies are required to determine whether islet transplantation plays a positive role in endothelial function recovery and neural regeneration. Second, islet transplantation can also regulate C-peptide secretion. Moreover, combined treatment with C-peptide and insulin had a significant effect on diabetic neuropathy compared to insulin injection alone [32]. Thus, the relationship between C-peptide and DMED should be investigated. In addition, islet cells can also secrete some non-classical islet peptides, such as GLP-1, GIP, xenin, oxytocin secreted by pancreatic islet α cells, and PYY, NPY secreted by islet PP cells, and Urocontin3 secreted by islet β cells. These hormones can regulate the function of pancreatic β-cells and the secretion of insulin, and have great potential in the treatment of diabetic facets [33]. Among them, GLP-1 stimulates insulin secretion, inhibits glucagon secretion, reduces food intake, reduces appetite, delays gastric emptying, reduces body weight, and protects βcells from apoptosis. The American Diabetes Association (ADA) and the European Association for Diabetes Study (EASD), recommend GLP1 agonists as adjunctive agents for metformin when monotherapy fails to meet therapeutic goals. Weihao Wang et al.’s meta-analysis also demonstrated that combination therapy with GLP-1 and insulin can achieve ideal therapeutic effects on glycemic control, weight loss, and insulin dose reduction in patients with type 1 diabetes [34]. Therefore, islet transplantation may improve the effect of fibrosis in penile tissue of diabetic rats by secreting these hormones, which is worthy of further study. Finally, our data demonstrated that islet transplantation improves penile tissue fibrosis 12 weeks after diabetes induction. However, the effects of islet transplantation should be investigated for longer-term DMED.

## Conclusions

This was the first study to demonstrate that islet transplantation could promote penile SMC regeneration and restore penile erectile function in advanced-stage DMED rats by inhibiting the TGF-β1/Smad2/CTGF pathway.

## Abbreviations

APO: Apomorphine
CTGF: Connective tissue growth factor
DMED: Diabetes mellitus erectile dysfunction
ED: Erectile dysfunction
FDA: Fluorescein diacetate
IEQ: Total islet equivalents
PDE5: Phosphodiesterase type 5
PI: Propidium iodide
SMCs: Smooth muscle cells
STZ: Streptozotocin
TGF-β1: Transforming growthfactor beta 1
α-SMA: Alpha 2 smooth muscle actin

## References

[1] Zhang X, Yang B, Li N, Li H (2017). Prevalence and risk factors for erectile dysfunction in Chinese adult males. J Sex Med.

[2] Hatzimouratidis K, Hatzichristou D (2014). How to treat erectile dysfunction in men with diabetes: from pathophysiology to treatment. Curr Diab Rep.

[3] Malavige LS, Levy JC (2009). Erectile dysfunction in diabetes mellitus. J Sex Med.

[4] Lue TF (2000). Erectile dysfunction. N Engl J Med.

[5] Vickers MA, Satyanarayana R (2002). Phosphodiesterase type 5 inhibitors for the treatment of erectile dysfunction in patients with diabetes mellitus. Int J Impot Res.

[6] Angulo J, Gonzalez-Corrochano R, Cuevas P, Fernandez A, La Fuente JM, Rolo F, Allona A, De Tejada IS (2010). Diabetes exacerbates the functional deficiency of NO/cGMP pathway associated with erectile dysfunction in human Corpus Cavernosum and penile arteries. J Sex Med.

[7] Gonzalez-Cadavid NF (2009). Mechanisms of penile fibrosis. J Sex Med.

[8] Verrecchia F, Mauviel A (2002). Control of connective tissue gene expression by TGF beta: role of Smad proteins in fibrosis. Curr Rheumatol Rep.

[9] Cho SY, Chai JS, Lee SH, Park K, Paick JS, Kim SW (2012). Investigation of the effects of the level of glycemic control on erectile function and pathophysiological mechanisms in diabetic rat. J Sex Med.

[10] Kwon O, Cho SY, Paick JS, Kim SW (2017). Effects of the start time of glycemic control on erectile function in streptozotocin-induced diabetic rats. Int J Impot Res.

[11] Choi WS, Kwon OS, Cho SY, Paick JS, Kim SW (2015). Effect of chronic administration of PDE5 combined with glycemic control on erectile function in streptozotocin-induced diabetic rats. J Sex Med.

[12] Lundberg J, Stone-Elander S, Zhang XM, Korsgren O, Jonsson S, Holmin S (2014). Endovascular method for transplantation of insulin-producing cells to the pancreas parenchyma in swine. Am J Transplant.

[13] Sutherland DER (2003). Current status of beta-cell replacement therapy (pancreas and islet transplantation) for treatment of diabetes mellitus. Transplant Proc.

[14] Pepper AR, Pawlick R, Gala-Lopez B, Macgillivary A, Mazzuca DM, White DJG, Toleikis PM, Shapiro AMJ, Diabetes I (2015). Reversed in a murine model by marginal mass syngeneic islet transplantation using a subcutaneous cell pouch device. Transplantation.

[15] He YQ, Xu ZQ, Zhou MS, Wu MM, Chen XH, Wang SL, Qiu KY, Cai Y, Fu HX, Chen BC, Zhou MT (2016). Reversal of early diabetic nephropathy by islet transplantation under the kidney capsule in a rat model. J Diab Res.

[16] Usuelli V, La Rocca E (2015). Novel therapeutic approaches for diabetic nephropathy and retinopathy. Pharmacol Res.

[17] Fensom B, Harris C, Thompson SE, Al Mehthel M, Thompson DM (2016). Islet cell transplantation improves nerve conduction velocity in type 1 diabetes compared with intensive medical therapy over six years. Diabetes Res Clin Pract.

[18] Zmuda EJ, Powell CA, Hai T. A method for murine islet isolation and subcapsular kidney transplantation. J Vis Exp. 2011;50 10.3791/2096 PMC3169267 21525838

[19] Heaton JP, Varrin S, Morales A (1991). The characterization of a bioassay of erectile function in a rat model. J Urol.

[20] Carson CC, Burnett AL, Levine LA, Nehra A (2002). The efficacy of sildenafil citrate (Viagra) in clinical populations: an update. Urology.

[21] Cellek S, Foxwell NA, Moncada S (2003). Two phases of nitrergic neuropathy in streptozotocin-induced diabetic rats. Diabetes.

[22] Ruan Y, Li M, Wang T, Yang J, Rao K, Wang S, Yang W, Liu J, Ye Z (2016). Taurine supplementation improves erectile function in rats with Streptozotocin-induced type 1 diabetes via amelioration of penile fibrosis and endothelial dysfunction. J Sex Med.

[23] Zhou F, Xin H, Liu T, Li GY, Gao ZZ, Liu J, Li WR, Cui WS, Bai GY, Park NC, Xin ZC (2012). Effects of icariside II on improving erectile function in rats with streptozotocin-induced diabetes. J Androl.

[24] Wei AY, He SH, Zhao JF, Iiu Y, Liu Y, Hu YW, Zhang T, Wu ZY (2012). Characterization of corpus cavernosum smooth muscle cell phenotype in diabetic rats with erectile dysfunction. Int J Impot Res.

[25] Sattar AA, Wespes E, Schulman CC (1994). Computerized measurement of penile elastic fibres in potent and impotent men. Eur Urol.

[26] Gray MA, Wang CC, Sacks MS, Yoshimura N, Chancellor MB, Nagatomi J (2008). Time-dependent alterations of select genes in streptozotocin-induced diabetic rat bladder. Urology.

[27] Mostafa ME, Senbel AM, Mostafa T (2013). Effect of chronic low-dose Tadalafil on penile cavernous tissues in diabetic rats. Urology.

[28] Massague J, Chen YG (2000). Controlling TGF-beta signaling. Genes Dev.

[29] Yamagishi S, Inagaki Y, Okamoto T, Amano S, Koga K, Takeuchi M (2003). Advanced glycation end products inhibit de novo protein synthesis and induce TGF-beta overexpression in proximal tubular cells. Kidney Int.

[30] Zhang LW, Piao SG, Choi MJ, Shin HY, Jin HR, Kim WJ, Song SU, Han JY, Park SH, Mamura M, Kim SJ, Ryu JK, Suh JK (2008). Role of increased penile expression of transforming growth factor-beta 1 and activation of the smadsignaling pathway in erectile dysfunction in streptozotocin-induced diabetic rats. J Sex Med.

[31] Zhou F, Li GY, Gao ZZ, Liu J, Liu T, Li WR, Cui WS, Bai GY, Xin ZC (2012). The TGF-beta1/Smad/CTGF pathway and corpus cavernosum fibrous-muscular alterations in rats with streptozotocin-induced diabetes. J Androl.

[32] Johansson BL, Borg K, Fernqvist-Forbes E, Kernell A, Odergren T, Wahren J (2000). Beneficial effects of C-peptide on incipient nephropathy and neuropathy in patients with type 1 diabetes mellitus. Diabet Med.

[33] Khan D, Moffet CR, Flatt PR, Kelly C (2018). Role of islet peptides in beta cell regulation and type 2 diabetes therapy. Peptides.

[34] Li R, Li Y, Y W, Zhao Y, Chen H, Yuan Y, K X, Zhang H, Y L, Wang J, Li X, Jia X, Xiao J (2018). Heparin-Poloxamer thermosensitive hydrogel loaded with bFGF and NGF enhances peripheral nerve regeneration in diabetic rats. Biomaterials.

